# Low-Magnitude High-Frequency Vibration Accelerated the Foot Wound Healing of n5-streptozotocin-induced Diabetic Rats by Enhancing Glucose Transporter 4 and Blood Microcirculation

**DOI:** 10.1038/s41598-017-11934-2

**Published:** 2017-09-14

**Authors:** Caroline Oi-Ling Yu, Kwok-Sui Leung, Jonney Lei Jiang, Tina Bai-Yan Wang, Simon Kwoon-Ho Chow, Wing-Hoi Cheung

**Affiliations:** 1 Department of Orthopaedics and Traumatology, Prince of Wales Hospital, The Chinese University of Hong Kong, Hong Kong SAR, China; 2 School of Biomedical Sciences, The Chinese University of Hong Kong, Hong Kong SAR, China; 3 The CUHK-ACC Space Medicine Centre on Health Maintenance of Musculoskeletal System, The Chinese University of Hong Kong Shenzhen Research Institute, Shenzhen, PR China

## Abstract

Delayed wound healing is a Type 2 diabetes mellitus (DM) complication caused by hyperglycemia, systemic inflammation, and decreased blood microcirculation. Skeletal muscles are also affected by hyperglycemia, resulting in reduced blood flow and glucose uptake. Low Magnitude High Frequency Vibration (LMHFV) has been proven to be beneficial to muscle contractility and blood microcirculation. We hypothesized that LMHFV could accelerate the wound healing of n5-streptozotocin (n5-STZ)-induced DM rats by enhancing muscle activity and blood microcirculation. This study investigated the effects of LMHFV in an open foot wound created on the footpad of n5-STZ-induced DM rats (DM_V), compared with no-treatment DM (DM), non-DM vibration (Ctrl_V) and non-DM control rats (Ctrl) on Days 1, 4, 8 and 13. Results showed that the foot wounds of DM_V and Ctrl_V rats were significantly reduced in size compared to DM and Ctrl rats, respectively, at Day 13. The blood glucose level of DM_V rats was significantly reduced, while the glucose transporter 4 (GLUT4) expression and blood microcirculation of DM_V rats were significantly enhanced in comparison to those of DM rats. In conclusion, LMHFV can accelerate the foot wound healing process of n5-STZ rats.

## Introduction

Type 2 diabetes mellitus (DM) is a metabolic disease where skeletal muscles have insulin resistance^1^. Delayed wound healing is a type 2 DM complication with diminished peripheral blood flow^2^ and impaired growth factor production^3^. DM foot ulcer is a presentation of delayed wound healing that affects more than 25% out of 150 million DM patients worldwide^4^, and can lead to lower limb amputations^5^.

DM wound is characterized by a disorder in the inflammatory and proliferative phases of the wound healing process^6^. Hyperglycemia contributes to delayed wound healing by exhibiting elevated levels of pro-inflammatory cytokines and proteases, which reduce levels of various growth factors^7^ and blood flow, diminish cell migration and impairs angiogenesis response, cell proliferation and reepithelialization in wounds^8^. It is important to accelerate the DM wound healing process by enhancing blood flow, cell proliferation and migration, and angiogenesis in wound, and enhancing glucose uptake in muscles to reduce the blood glucose level.

Exercise is a well-established method to control the blood glucose level and is the main strategy to treat type 2 DM patients^1^. However, DM patients with complications may have difficulties in regular daily exercise. Low magnitude high frequency vibration (LMHFV) has been proven as an alternative to conventional exercise that can stimulate metabolic responses^9^. In clinical and preclinical studies, LMHFV has been reported to have benefits on muscle contractibility functions^10^, muscle structures and strength^11^. It has also been proven to be effective in inducing tissue regeneration, and promoting blood flow^12^. Ten minutes of vibration treatment can significantly increase the blood flow of healthy adults between 18–43 years old^13^. Non-DM patients have also shown reduced blood glucose levels after vibration treatments^14^. These are the essential criteria in accelerating a DM wound healing process.

In this study, we hypothesized that LMHFV could accelerate the foot wound healing process of n5-STZ-induced DM rats by enhancing skeletal muscle activities to increase glucose uptake, reduce blood glucose, and enhance blood microcirculation to increase cell proliferation and angiogenesis. The objective was to investigate the effects of LMHFV on the DM foot wound healing process using neonatal n5-STZ-induced DM rats by comparing with no-treatment DM rats (DM) and non-DM rats at Days 1, 4, 8 and 13 post wounding.

## Results

### Rat Physical Health

DM rats increased their food and water uptakes beginning at week 5 after birth. The average blood glucose level of DM rats in both groups before open-wound induction was about 483.47 ± 91.46 mg/dL, while those of non-DM rats was about 106.6 ± 1.8 mg/dL.

### Wound Morphology

**Figure 1:**
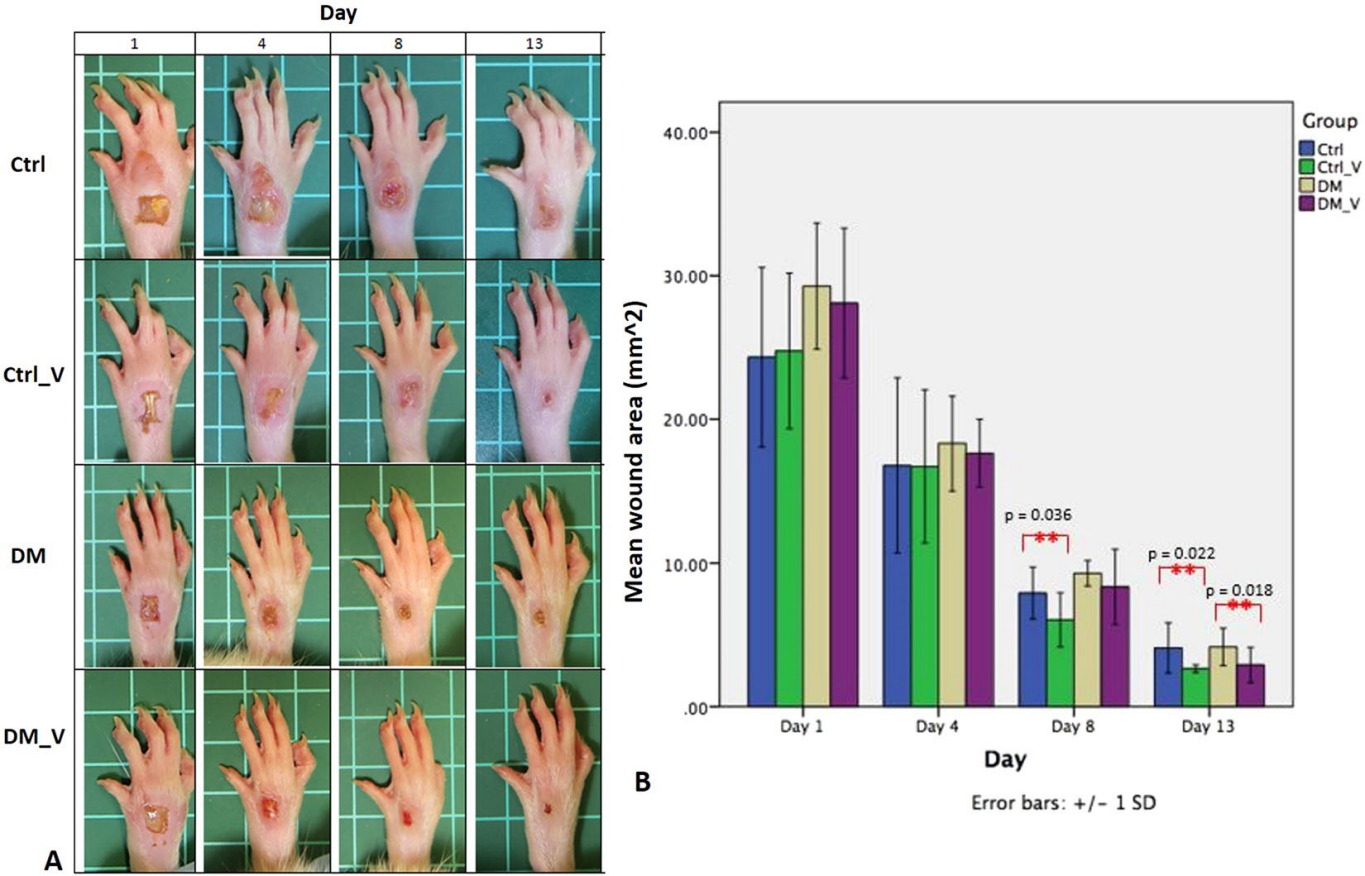
Images of the foot wounds of at Days 1, 4, 8, and 13 post wounding (n = 6/time point). (**A**) The wound size of all groups stretched far apart due to edema and swelling expanded to the whole foot at Day 1 post wounding. Swelling at the foot wound of DM rats continued at Day 13, but the DM_V rats subsided at Day 8. (**B**) At Day 13, the wound size area of the DM_V rats was significantly smaller than those of the DM rats. The wound size of the Ctrl_V rats were significantly smaller than those in Ctrl rats at Days 8 and 13 post wounding. At Day 8, the wound size area of the DM rats was significantly larger than that in the Ctrl rats. **Significant difference.

Wounds created (Day 0) were consistent in size and shape with minimal to no bleeding in all groups. The wound size of all groups were increased at Day 1, as the edges of the wounds were stretched further apart due to edema and swelling on the whole foot Fig. 1A. Swelling at the foot wound continued in DM rats at Day 13, but subsided in DM_V rats at Day 8. At Day 13, the wound size of the DM_V rats (2.90 ± 1.23 mm^2^) was significantly smaller (p = 0.018) than the DM rats (4.16 ± 1.30 mm^2^). The wound area of the Ctrl_V rats (Day 8, 6.04 ± 1.88 mm^2^; Day 13, 2.64 ± 0.26 mm^2^) were significantly smaller than Ctrl rats (Day 8, 7.91 ± 1.80 mm^2^; Day 13, 4.09 ± 1.75 mm^2^) (p = 0.036 and 0.022, respectively). Swelling at the foot wound of Ctrl rats subsided as early as Day 4. At Day 8, the wound area of the DM rats (9.29 ± 0.88 mm^2^) was significantly larger (p = 0.048) than that in the Ctrl rats (7.91 ± 1.80 mm^2^) Figs 1B and 8.

### Blood Glucose Level

**Figure 2:**
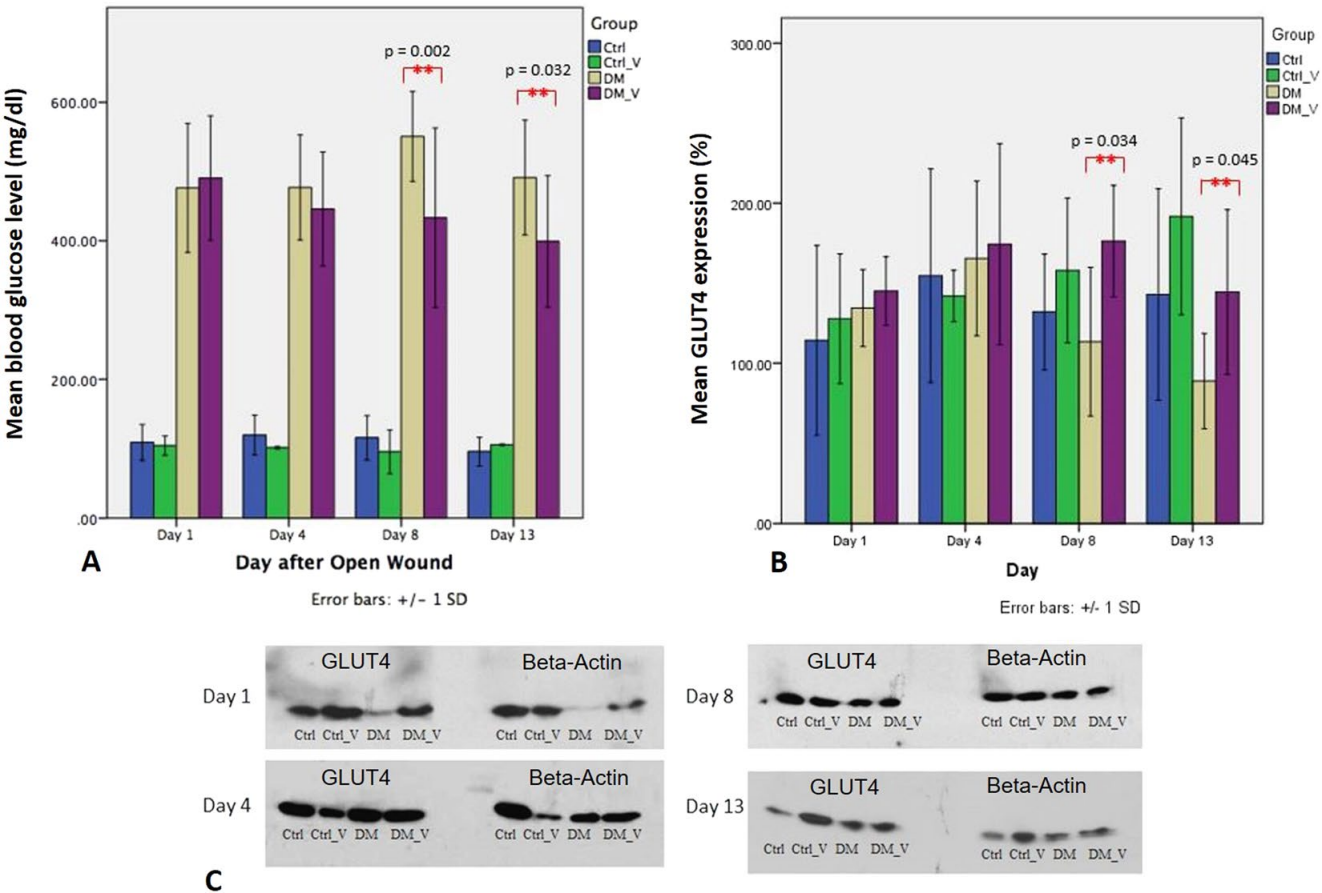
Comparison of the blood glucose level and GLUT4 expressions extracted from Western Blot between the Ctrl, Ctrl_V, DM, and DM_V groups (n = 6/time point). (**A**) The blood glucose levels of DM_V rats were significantly lower than that observed on DM rats at Day 8 and 13 post wounding. No significant differences were observed between Ctrl_V and Ctrl rats. (**B**) The glucose transporter 4 (GLUT4) expressions of DM_V rats were significantly higher than that observed on DM rats at Day 8 and 13 post wounding. No significant differences were observed between Ctrl_V and Ctrl rats. (**C**) Cropped blots of GLUT4 and Beta-Actin are displayed in comparison between the Ctrl, Ctrl_V, DM, and DM_V groups. **Significant difference.

The blood glucose level of DM_V rats (Day 8, 433.20 ± 129.74 mg/dl; Day 13, 399.15 ± 94.9 mg/dl) was significantly lower than those observed on DM rats (Day 8, 550.76 ± 64.93 mg/dl; Day 13, 491.26 ± 82.96 mg/dl) (p = 0.002 and 0.032, respectively) Fig. 2A. No significant differences were observed between Ctrl_V and Ctrl rats Fig. 8.

### Glucose Transporter 4 (GLUT4) Immunoblotting

The GLUT4 expressions of DM_V rats (Day 8, 176.30 ± 34.88%; Day 13, 144.48 ± 51.44%) were significantly higher than those observed on DM rats (Day 8, 113.39 ± 46.45%; Day 13, 88.78 ± 29.78%) (p = 0.034 and 0.045, respectively) Fig. 2B. No significant difference was observed between Ctrl_V and Ctrl rats Fig. 8.

### Blood Perfusion by Laser Doppler Measurement

**Figure 3:**
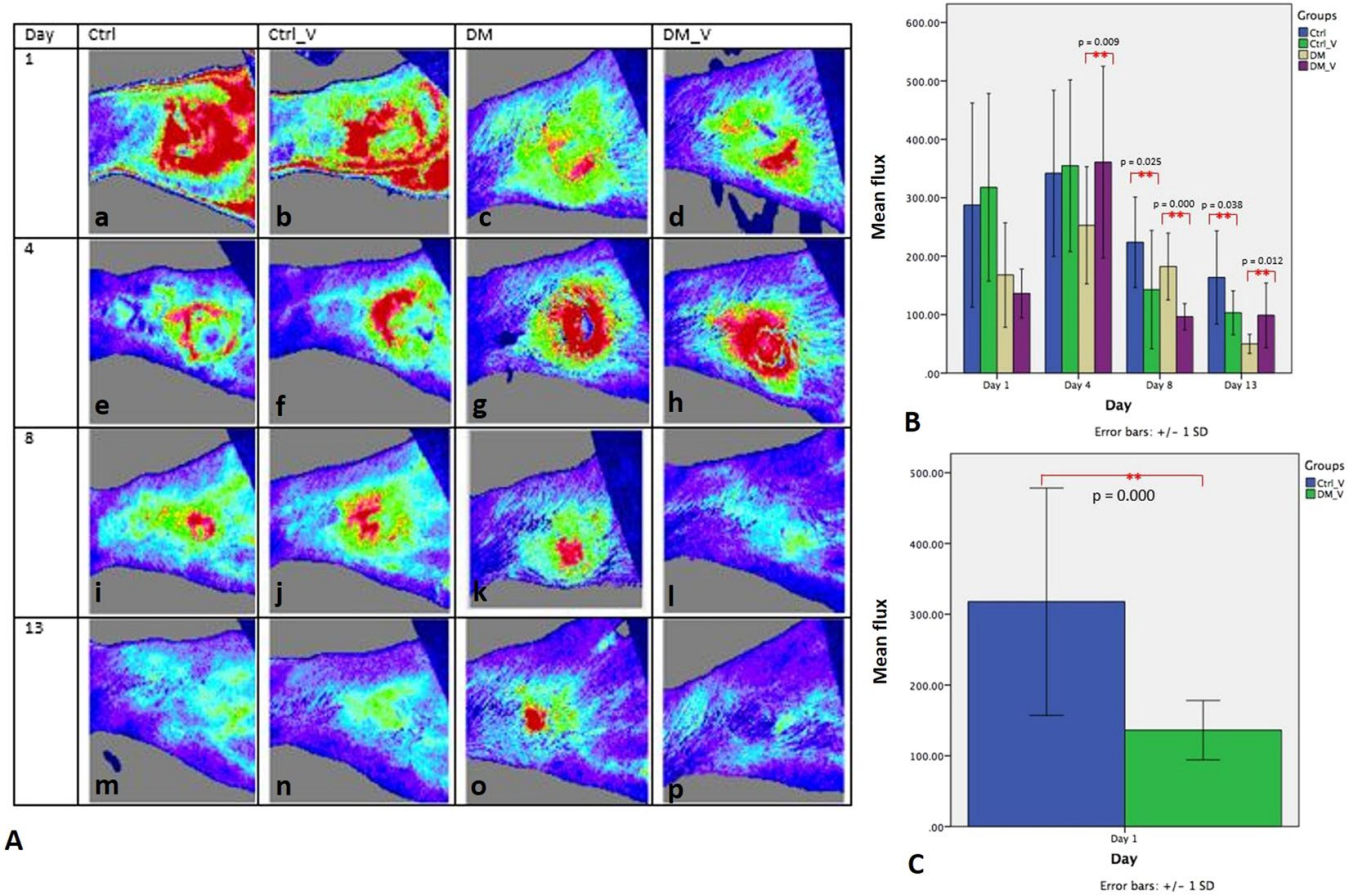
Comparison of the laser Doppler imaging measurement (Flux) between the Ctrl, Ctrl_V, DM, and DM_V groups (n = 6/time point). (**A**) Images of flux of all groups. DM_V rats had more blood circulation (higher flux) at Day 1 post wounding, and inflammation slowly subsided at Day 4 and reduced at Day 13 post wounding. Inflammation in the wounds of DM rats persisted until Day 13 post wounding. (**B**) The flux of DM_V rats were significantly higher than those in DM rats at Days 4 and 13 post wounding. The flux of Ctrl_V rats were significantly lower than those in Ctrl rats at Days 8 and 13 post wounding. At Day 1, the flux of DM_V rats was significantly lower than those of Ctrl_V rats. (**C**) Ctrl_V rats had significantly higher flux than those of DM_V rats. **Significant difference.

A laser Doppler imaging device was used to evaluate the blood flow of the wound in flux value Fig. 3A. The flux of DM_V rats (Day 4: 360.91 ± 164.04%; Day 13: 98.72 ± 55.33%) was significantly higher than those in DM rats (Day 4: 252.83 ± 100.24%; Day 13: 49.89 ± 16.46) (p = 0.009 and 0.012, respectively). The flux of Ctrl_V rats (Day 8: 142.73 ± 101.35%; Day 13: 103.13 ± 37.66%) was significantly lower than those in Ctrl rats (Day 8: 223.76 ± 77.38%; Day 13: 163.63 ± 79.83%) (p = 0.025 and 0.038, respectively). At Day 1, the flux of DM_V rats (136.16 ± 41.93) was significantly lower than those in Ctrl_V rats (317.70 ± 160.61) (p < 0.05) Fig. 3B. Comparing DM and non-DM rats, Ctrl_V rats had significantly higher flux than those of DM_V rats Figs 3C and 8.

### Histology

**Figure 4:**
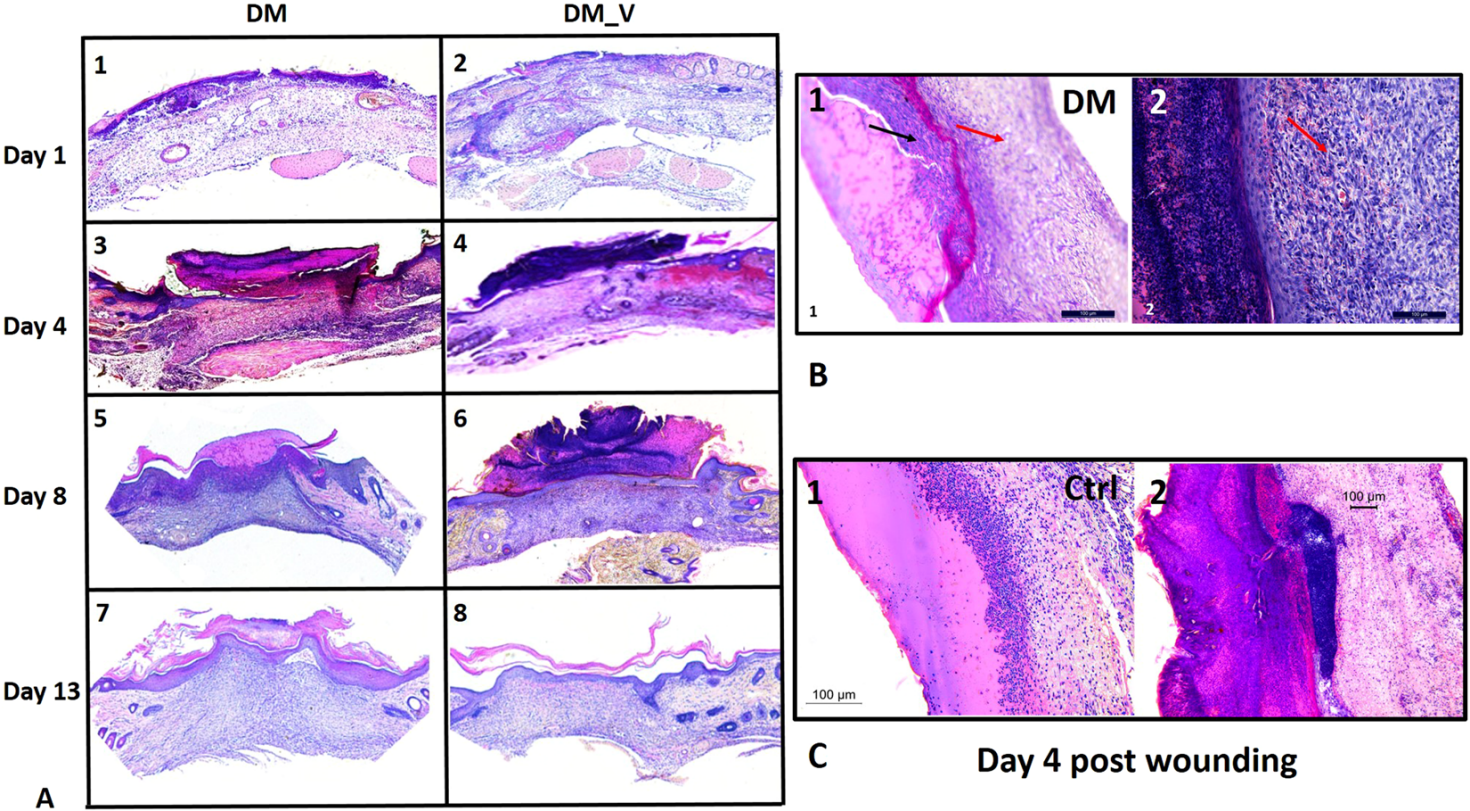
Representative histology images. (**A**) H&E staining at 5X showed granulation formation in the foot wounds of DM_V rats at Day 8 post wounding. (**B**) The H&E images at 20X. Red arrows indicated the formation of granulation tissues below the surface of the wound. Black arrow in B1 indicated inflammation in the wounds of DM rats that are absence in the wounds of DM_V rats (B2). (**C**) The H&E images at 20X showed the epithelial layer of Ctrl_V rats healed faster at Day 4 post wounding by forming crust/scab on the surface of the wound, while inflammation was still observed in the epithelial layer of Ctrl rats.

At Day 1, a new epithelium was observed on the surface of the wound for both DM groups. By Day 4, the epithelial layer of DM_V rats was more complete than DM rats. A crust or scab was observed on the wound surface of the DM_V rats. By Day 8, more granulation tissue was observed in DM_V rats than those of DM rats Fig. 4.

### Expression of PECAM-1

**Figure 5:**
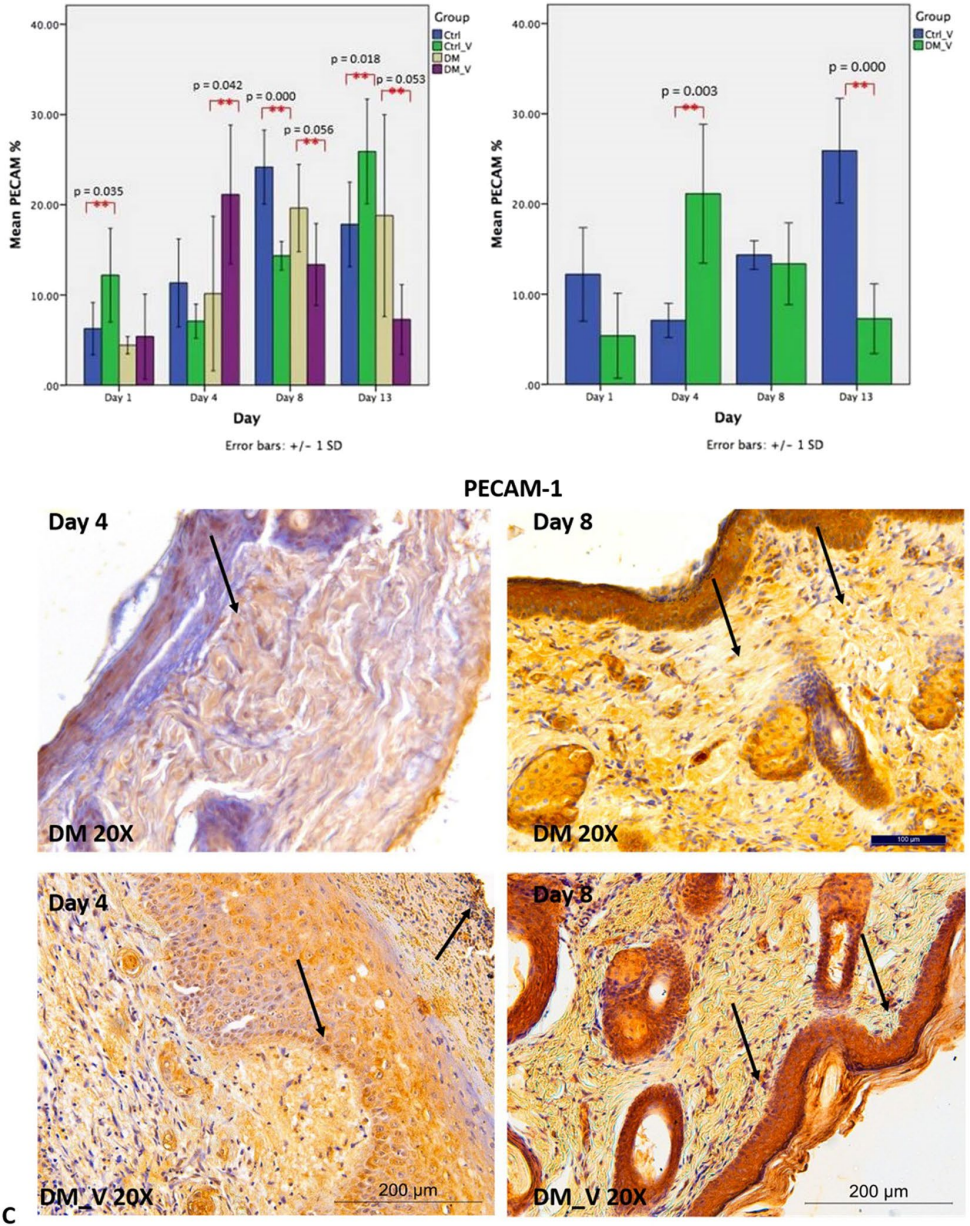
Comparison of PECAM-1 immunohistochemistry between the Ctrl, Ctrl_V, DM, and DM_V groups (n = 6/time point). (**A**) PECAM-1 expression of DM_V rats was significantly higher than those of DM rats at Day 4 post wounding. PECAM-1 expression of DM_V rats showed lower than those of DM rats generally, with significant differences at Days 8 and Day 13. PECAM-1 of Ctrl_V rats was significantly higher than those of Ctrl rats, but Ctrl_V rats was significantly lower than those of Ctrl rats at Day 8. (**B**) Comparing between vibration groups, PECAM-1 of DM_V rats was significantly higher than those in Ctrl_V rats at Day 4 post wounding, but significantly lower by Day 13. (**C**) Image of DM_V at 20X magnification. **Significant difference. Note: Arrows indicate PECAM-1 expression.

PECAM-1 was performed to evaluate the presence of endothelial cells in the wound. PECAM-1 of DM_V rats (21.13 ± 7.69%) was significantly higher (p = 0.042) than those of DM rats (10.15 ± 8.56%) at Day 4. By Days 8 and 13, PECAM-1 of DM_V rats (Day 8, 13.37 ± 4.53%; Day 13, 7.28 ± 3.86%) was significantly lower than those of DM rats (Day 8, 19.63 ± 4.84%; Day 13, 18.80 ± 11.19%) (p = 0.056, and 0.053, respectively) Fig. 5A. PECAM-1 of Ctrl_V rats (Day 1, 12.19 ± 5.19%; Day 13, 25.90 ± 5.79%) were significantly higher than those of Ctrl rats (Day 1, 6.25 ± 2.89%; Day 13, 17.81 ± 3.44%) (p = 0.035 and 0.018, respectively), but Ctrl_V rats (14.34 ± 1.57%) was significantly lower (p = 0.000) than those of Ctrl rats (24.17 ± 4.09%) at Day 8. Comparing between vibration groups, PECAM-1 of DM_V rats (21.13 ± 7.69%) was significantly higher (p = 0.03) than those of Ctrl_V rats (7.08 ± 1.88%) at Day 4, but significantly lower (p < 0.05) by Day 13 Figs 5B and 8.

### Expression of VEGF

**Figure 6:**
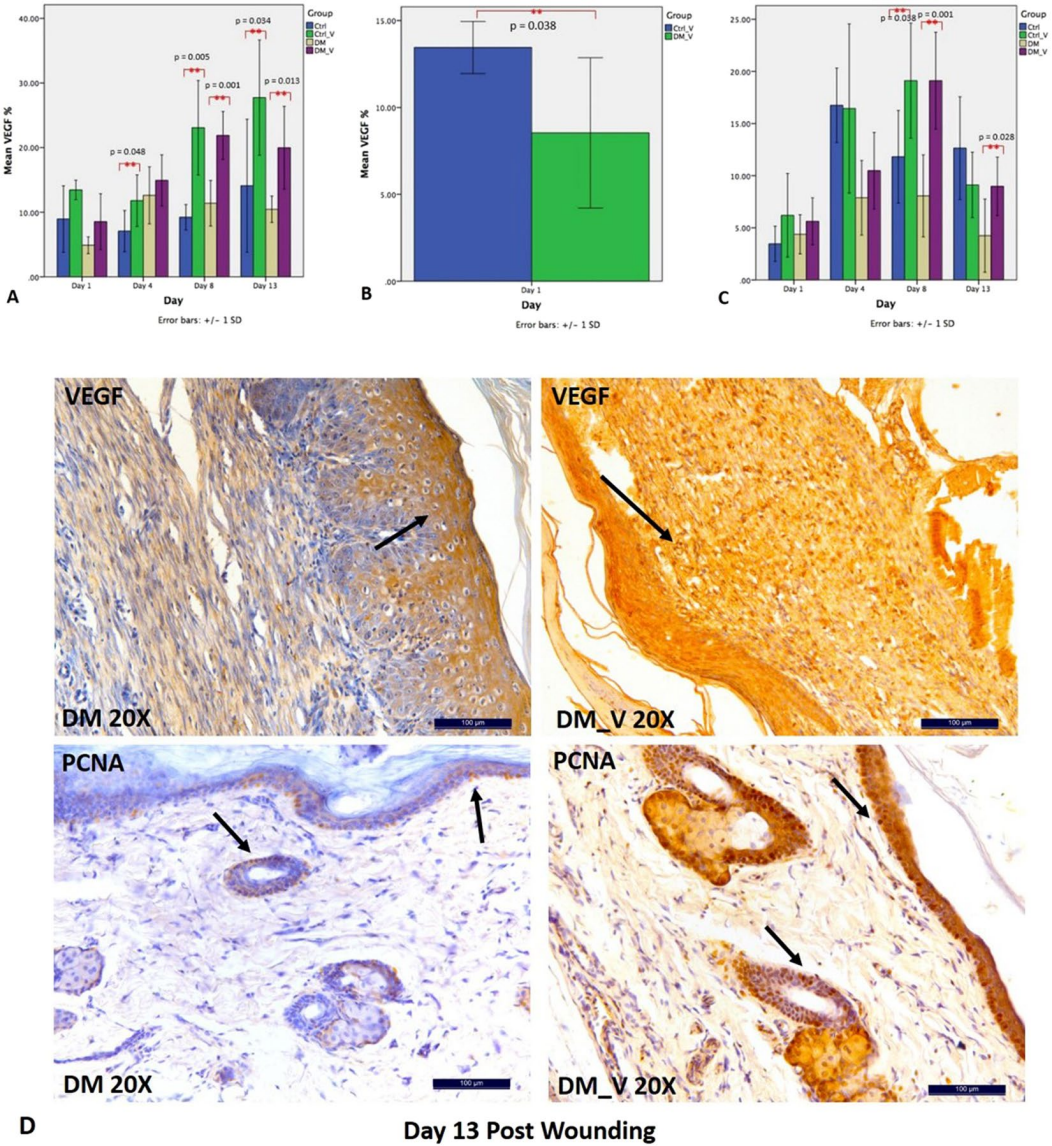
Comparison of PCNA and VEGF immunohistochemistry between Ctrl, Ctrl_V, DM, and DM_V groups (n = 6/timpoint). (**A**) VEGF of DM_V rats were significantly higher than those of DM rats at Days 8 and 13 post wounding. VEGF of Ctrl_V rats were significantly higher than those of Ctrl rats at Days 4, 8, and 13 post wounding. (**B**) Comparing between vibration groups, VEGF of Ctrl_V rats was significantly higher than those of DM_V rats at Day 1. (**C**) PCNA of DM_V rats were significantly higher than those of DM rats at Days 8 and 13. PCNA on Ctrl_V rats was significantly higher than those of Ctrl rats at Day 8. **Significant difference. Note: Arrows indicate PCNA and VEGF expressions.

VEGF was performed to evaluate angiogenesis^15^. VEGF of DM_V rats (Day 8, 21.80 ± 3.70%; Day 13, 19.97 ± 6.40) were significantly higher than those of DM rats (Day 8, 11.38 ± 3.53%; Day 13, 10.45 ± 2.04%) (p = 0.001 and 0.013, respectively) Fig. 6A. VEGF of Ctrl_V rats (Day 4, 11.78 ± 3.99%; Day 8, 23.06 ± 7.3%; Day 13, 27.73 ± 8.91%) were significantly higher than those of Ctrl rats (Day 4, 7.07 ± 3.18%; Day 8, 9.21 ± 1.96%; Day 13, 14.09 ± 10.29%) (p = 0.048, 0.005, and 0.034, respectively). Comparing between vibration groups, VEGF of Ctrl_V rats (15.44 ± 1.5%) was significantly higher than those of DM_V rats (8.53 ± 4.32%) at Day 1 (p = 0.038) Figs 6B and 8.

### Expression of PCNA

PCNA was performed to evaluate cell proliferations. PCNA of DM_V rats (Day 8, 19.22 ± 4.63%; Day 13, 8.97 ± 2.80%) were significantly higher than those of DM rats (Day 8, 8.06 ± 3.93%; Day 13, 4.24 ± 3.50%) (p = 0.001 and 0.028, respectively) Fig. 6C. PCNA on Ctrl_V rats (19.11 ± 4.63%) was significantly higher (p = 0.038) than those of Ctrl rats (11.81 ± 4.44%) at Day 8 Fig. 8.

### C-Reactive Protein (CRP)

**Figure 7:**
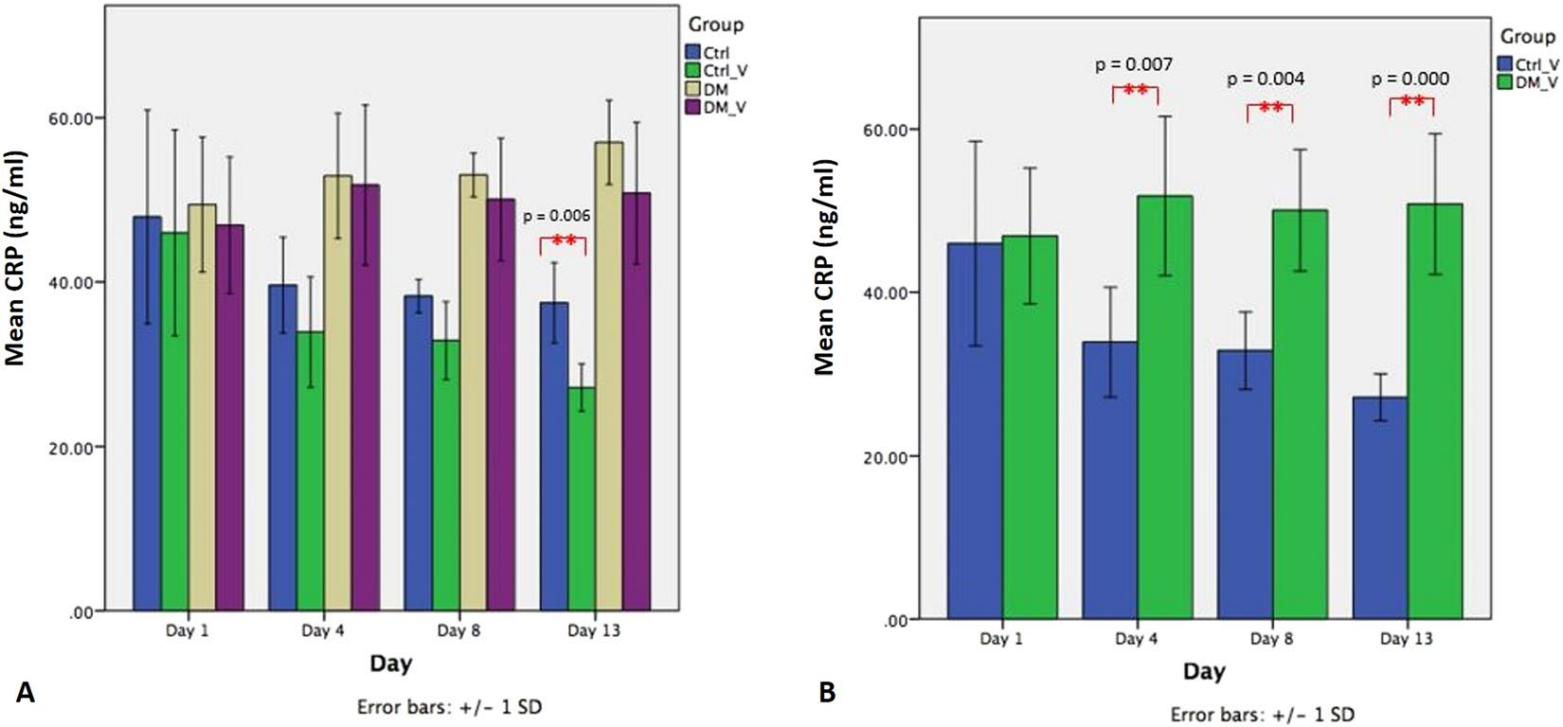
Comparison of the C-Reactive protein level of Ctrl, Ctrl_V, DM, and DM_V groups (n = 6/time point). (**A**) The CRP of Ctrl_V rats was significantly lower (p = 0.006) than those of Ctrl rats at Day 13 post wounding. No significant difference observed between DM_V and DM rats. (**B**) Comparing between vibration groups, the CRP levels of DM_V rats were significantly higher than those of Ctrl_V rats at Days 4, 8 and 13 post wounding. **Significant difference.

**Figure 8:**
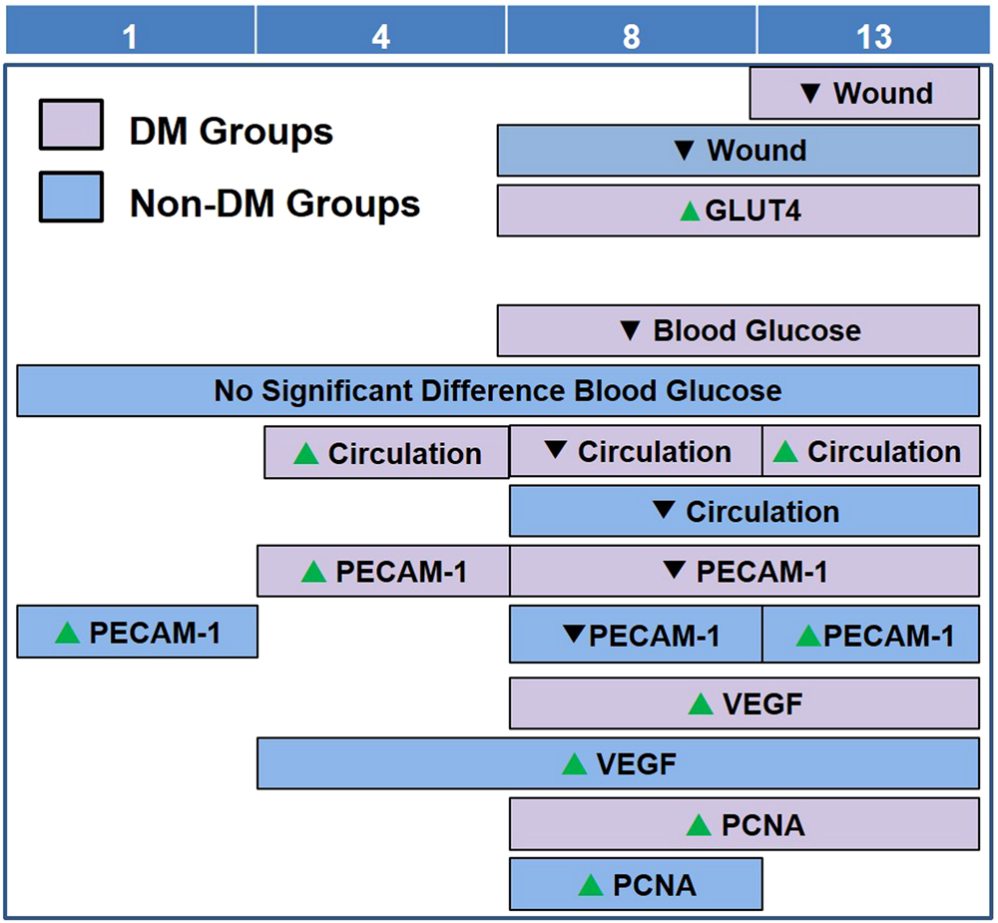
Summary of significant differences among all groups in different assessments at Day 1, 4, 8, and 13 post-wounding. Purple boxes represent significant difference between DM_V rats and DM rats. Blue boxes represent significant difference between Ctrl_V rats and Ctrl rats. Green arrows represent significant increase, while black arrows represent significant decrease.

CRP was used as a marker to compare systemic inflammation between groups Fig. 7A. The CRP of Ctrl_V rats (27.17 ± 2.85 ng/ml) was significantly lower (p = 0.006) than those of Ctrl rats (37.46 ± 4.88 ng/ml) at Day 13. The CRP levels of DM rats (Day 4, 52.92 ± 7.62 ng/mL; Day 8, 53.03 ± 2.66 ng/mL) were significantly higher than those of Ctrl rats (Day 4, 39.60 ± 5.83ng/mL; Day 8, 38.28 ± 2.01 ng/mL) (p = 0.007 and 0.000, respectively). Comparing between vibration groups, the CRP levels of DM_V rats (Day 4, 51.80 ± 9.75 ng/mL; Day 8, 50.05 ± 7.45 ng/mL; Day 13, 50.81 ± 8.62 ng/mL) were significantly higher than those of Ctrl_V rats (Day 4, 33.91 ± 6.70 ng/mL; Day 8, 32.87 ± 4.71 ng/mL; Day 13, 27.17 ± 2.85 ng/mL) (p = 0.007, 0.004, and 0.000, respectively) Figs 7B and 8.

## Discussions

This study investigates the effects of LMHFV on the foot wound healing process using n5-STZ-induced DM rats. Our results revealed that LMHFV accelerated the foot wound healing process in both DM (DM_V) and non-DM rats (Ctrl_V). The foot wounds of the DM_V rats healed significantly faster by LMHFV compared to DM rats at Day 13, and Ctrl_V rats healed even faster with significant differences at Days 8 and 13 than those of Ctrl rats. LMHFV also significantly enhanced GLUT4 expression by stimulating muscle activities of DM_V rats to increase glucose uptake and to reduce the blood glucose level. Blood microcirculation was also improved in both DM (DM_V) and non-DM (Ctrl_V) rats to enhance cell proliferation, angiogenesis, and local inflammatory response in wounds Fig. 8. These evidence of enhanced muscle activities and blood microcirculation by LMHFV indicate the significance in accelerating DM wound healing.

LMHFV had enhanced the muscle activities of DM_V rats by significantly increasing their GLUT4 expression for glucose uptake and reducing their blood glucose level at Days 8 and 13. According to Stewart *et al*., type IIa fibers contract at the rates of 20–70 Hz^16^ and our previous studies also showed these fibers were more sensitive to LMHFV than other muscle fiber types in the gastrocnemius to improve muscle functions^11^. Since approximately 22% of gastrocnemius is composed of type IIa fibers^17^, LMHFV had enhanced muscle activities by triggering type IIa fibers to increase GLUT4 expression for glucose uptake. Hussey *et al*. observed that a single bout of acute exercise on a cycle ergometer for an hour increased GLUT4 in skeletal muscles of type 2 DM patients by increasing AMPK and p38 MPK phosphorylation^18^. Since Hargreaves *et al*. had demonstrated that glucose uptake was enhanced by facilitated diffusion after increasing blood flow in contracting skeletal muscle^19^, our flux data further supported that LMHFV enhanced glucose uptake after increasing blood flow systemically. Del Pozo-cruz *et al*. also showed vibration could significantly reduce HbA1c and fasting blood glucose in DM patients after a 12-week vibration treatment^20^, indicating that enhanced muscle activities can indirectly reduce the blood glucose level. Studies have shown that exercise could enhance glucose uptake in type 2 DM patients with different signaling pathways^1^, but the exact mechanism on which LMHFV triggers type IIa fibers to release GLUT4 expression and increase glucose uptake needs further study to clarify.

LMHFV had significantly enhanced blood microcirculation to increase PECAM-1 during the inflammatory phase, and PCNA and VEGF during the proliferative and angiogenesis phases to accelerate wound healing. Weinheimer-Haus *et al*. reported that low intensity vibration (LIV) at 45 Hz, 0.4 g for 30 min/day for 5 days/week could accelerate wound healing in mice by increasing blood flow to enhance angiogenesis and granulation tissue formation/re-epithelialization at day 7 post wounding^4^. In our previous published paper, we have used both 3-D high-frequency power Doppler and micro-computed tomography (microCT) microangiography to demonstrate that LMHFV could increase blood flow and angiogenesis in both normal and osteoporotic fractures at the femora^21^. Our data had indicated that LMHFV has significantly enhanced the skin blood microcirculation (Day 4 and 13) and VEGF (Day 8) of DM_V rats more than those of DM rats. In the inflammatory phase, PECAM-1 recruits leukocytes at inflammatory sites and across the endothelial layer^22^. Since PECAM-1 is an indicator for the presence of endothelial cells in the wound, LMHFV might have significantly enhanced PECAM-1 through shear stress on endothelial cells to modulate inflammatory signaling pathways^23^. PECAM-1 was also significantly reduced after leukocytes emigrated^24^. This supports our data that PECAM-1 was significantly enhanced at Day 4 and reduced at Days 8 and 13 in DM_V rats. Furthermore, PCNA of DM_V rats was also enhanced at Days 8 and 13. Since PCNA is an indicator of epidermal proliferation and granulation tissue formation, LMHFV might have also induced mechanical stress in DM_V rats to create an inflammatory response to enhance cellular proliferation for tissue repair^25^. Our histology images further showed that more granulation tissues were formed in the wounds of the DM_V rats than those of the DM rats since Day 8. Granulation formation is related to blood microcirculation since regions of granulation have a higher blood flow than those without granulation^26^. Thus, LMHFV might have promoted granulation tissue formation by enhancing blood microcirculation and angiogenesis in wound to accelerate wound healing. The blood microcirculation of Ctrl_V rats was significantly lower than those of Ctrl rats at Days 8 and 13. This might be because the wound size of Ctrl_V rats was also significantly reduced at the same time requiring lower blood flow to complete the wound healing process. Rendell *et al*. also reported that the blood flow in the paw wounds of non-DM rats was significantly reduced at Day 7 post wounding^27^. This indicates that the microvascular structure of the wound of Ctrl_V rats might be nearly completed to regulate blood microcirculation normally.

Comparing between DM and non-DM groups, all expressions of Ctrl_V rats were significantly different at an earlier wound healing process from those of DM_V rats. The PECAM-1 of Ctrl_V rats was significantly enhanced at Day 1 but reduced at Day 8 than DM_V rats. The VEGF of Ctrl_V rats were significantly higher than those of DM_V rats at Day 1. Ctrl_V rats had significantly higher PCNA than Ctrl rats at Day 8. This evidence further explained why the wound area of Ctrl_V rats healed earlier than DM_V rats.

LMHFV had little effect in reducing the CRP level in DM_V rats because it was positively associated with hyperglycemia independently^28^. Although the blood glucose level of DM_V rats was significantly reduced at Days 8 and Day 13, it remained high (>300 mg/dl) after a two-week vibration treatment. Thus, the CRP level may not be reduced until hyperglycemia is controlled. Increased platelet reactivity might be another explanation, since elevated CRP level in blood was observed in DM patients after exercise^29^. Comparing between DM and non-DM rats, the CRP level of Ctrl_V rats was significantly lower than those of DM_V rats at Days 4, 8, 13 post wounding. The CRP level of Ctrl_V rats was also significantly lower than those in Ctrl rats at Day 13. These observations indicated that inflammation was significantly reduced in non-DM rats since Day 4 post wounding, and LMHFV further reduced the CRP level in Ctrl_V rats.

The differences of tissue architectures and immune responses^30^ in wound healing between humans and rats are limitations to this study. The open wound model used is not equivalent to a human’s chronic non-healing wound^31^, but its characteristics match with the clinical observations of a DM foot ulcer to study the wound healing process of a DM wound. In western blot analysis, we only used one loading control to compare with GLUT4 expression. Although Vigelso *et al*. only recommended not using beta-actin as loading control if muscle samples are obtained from elderly or with a large age difference^32^ and multiple literatures also used beta-actin as the loading control^33, 34^, we could use another loading control such as GAPDH to double check its reliability. Furthermore, we only measured the wound area of each rat based on how its wound closed up at different time points but did not measure the wound depth of each rat because we lack the technique to measure them precisely in this study.

In conclusion, LMHFV accelerated an open foot wound healing process of n5-STZ-induced type 2 DM rats by enhancing muscle activities to increase glucose uptake and reduce the blood glucose level, and improving blood microcirculation to increase cell proliferation and angiogenesis. Non-DM rats with vibration treatments healed the fastest compared to other groups with earlier enhancement of wound healing factors including blood flow and cell proliferation. Due to hyperglycemia, the wounds of DM_V rats healed slower than Ctrl_V rats, but much faster than DM rats. Reduced blood glucose level of DM_V rats at Days 8 and 13 might explain the enhancement of VEGF, PCNA, and GLUT4 expressions at the same time. These indicators showed evidences that the wound healing process of DM_V rats was significantly accelerated by LMHFV. Therefore, LMHFV is recommended as an alternative non-invasive therapeutic device to accelerate the skin wound healing process in DM patients.

## Methods

### Animal Model and Study Design

A total of 96 female albino Wistar rats were equally divided into DM-Vibrated (DM_V), DM-Control (DM), Non-DM Vibrated (Ctrl_V), and Non-DM Control (Ctrl) groups (n = 24/group). Time points were as follows (n = 6/group/time point): Days 1, 4, 8, and 13^35^. In both DM groups, 5-day-old Wistar rats were intraperitoneally injected with streptozotocin (STZ, Sigma-Aldrich, 70 mg/kg) that was freshly dissolved in 0.1 M citrate buffer (pH 4.5). Rats were supplied by and kept in the Laboratory Animal Service Center of the Chinese University of Hong Kong (CUHK) and resided in a room with a 12-h light-dark cycle. Based on our established protocol, the DM status of STZ-induced rats was confirmed at 10-week old with a Contour Plus glucometer (Bayer Healthcare, Germany)^36^. Rats with a blood glucose level ≥300 mg/dL were randomized to either DM or DM_V group.

The animal experiments were conducted under a license issued by the Animals (control of experiments) Ordinance (Cap. 340) of the Department of Health of the Hong Kong government, and approval was obtained from the Animal Experimentation Ethics Committee (Ref: 13/085/GRF-5), CUHK. All experiments were performed in accordance with relevant guidelines and regulations.

### Foot Wound Creation

Induction of an open foot wound was based on an excisional model^35^ to produce more scar tissue for analysis^37^. In past DM foot ulcer/wound studies, the wound location was usually created on the back of the rats/mice^38–41^. Few papers induced a wound on plantar skin of the paw^42^ or at the dorsum of the foot^43^, but none of these models used type 2 DM rats/mice. Since 90% of DM patients worldwide are type 2 DM^6^ and approximately 5–7% of them will eventually have foot ulcer^44^, a clinically relevant animal model for research using an open foot wound model in type 2 DM rats is important^36^.

Nine weeks after STZ injection, a glucometer (Contour Plus, Bayer Healthcare, Germany) was used to determine the blood glucose level of each rat. Adult rats with a blood glucose level of ≥300 mg/dl were used. On the day of open wound induction (Day 0), each rat was anesthetized with 75 mg/kg ketamine and 10 mg/kg xylazine. The skin on the right footpad of the hindlimb was shaved and a 2 mm × 5 mm rectangular full thickness wound was created. The wound size used in this experiment was based on standard wound models^30^.

### Low Magnitude High Frequency Vibration (LMHFV) Treatment

LMHFV treatment was started at Day 1 post wounding, which the DM_V and Ctrl_V rats stood on a LMHFV platform (35 Hz, 0.3 g) for 20 min/day, 5days/week until endpoint^45^. DM and Ctrl rats also stood on the LMHFV platform with the same regime with the power off. Treatments were given in the morning from Monday to Friday for consecutively 5 days per week. The break was set at the weekends. The transmission of mechanical signal to hindlimbs have been validated and reported in our previous study^12^. The rats were placed on the platform by housing individually in a bottomless compartmented cages with their hindlimbs touching well to the platform, where the partitions were in black to minimize the surrounding disturbance.

### Wound Size Measurement

The wound area was measured with a photo imaging software (SPOT 3.5.5 Window)^36^. Before wound area measurement, the wound edges of the wound were defined by comparing the color difference in the wound area and those of nearby non-wounded skin area of the same rat. The color of the wounded skin and non-wounded skin were distinguished using the magnetic lasso tool of Photoshop CS6 software before using the photo imaging software (SPOT 3.5.5 Window) to calculate the wound area. Rat wounds and a metric ruler with standardized 1 cm × 1 cm squares were photographed simultaneously. Digital photographs of the injury site were taken using a Canon SX50HS digital camera. The wound size was calculated and measured using the software based on the standardized squares.

### Blood Glucose Level

Blood glucose was collected from the tail and analyzed with a Contour Plus glucometer (Bayer Healthcare, Germany)^35^. The blood glucose level of each rat was monitored at each time point until scarification.

### Glucose Transporter 4 (GLUT4) by Western Blot

Total protein of glucose transporter 4 (GLUT4) was extracted from gastrocnemius^46^. Each sample was homogenized and centrifuged at 11,000 g for 20 minutes. The supernatant was determined with the Pierce BCA Protein Assay Kit (Thermo Scientific, Massachusetts, USA). 40 μg proteins/sample was denatured and separated in 10% SDS-PAGE. Primary antibody GLUT4 (1F8) Mouse mAb (Cell Signaling Technology, Massachusetts, USA) was applied at 1:1000 dilution and incubated at 4 °C overnight. After incubation, the membranes were incubated with anti-mouse IgG, HRP-linked antibody (1:5000 diluted in TBST) at 37 °C for 60 min. The protein bands were visualized with Clarity Western ECL Blotting Substrates (Bio-Rad, California, USA), following the manufacturer’s guidelines and quantified using ImageJ software (NIH, Maryland, USA). β-Actin was used as reference protein.

### Blood Perfusion by Laser Doppler Imaging

The blood perfusion on the surface of the wound was measured with the laser Doppler imaging (Moor Instruments Ltd, UK). The wounded foot was scanned using the repeat image measurement mode. The unwounded foot of each rat was scanned as control. All data were quantified, in terms of flux, and automatically calculated using the Moor FLPI measurement software (Version 2.1)^47^.

### Histology

The new full-thickness skin layer that was regenerated at the wound site during post wounding was removed by a surgical scalpel for histological analyses^48^. Samples were obtained after animal euthanasia. Samples were fixed in 4% paraformaldehyde, embedded, and sectioned at 8 µm. The sections were stained with hematoxylin and eosin (H&E). Section images were captured with microscope (DM5000, Leica Microsystems Gmblt, Wetzlar, Germany) to evaluate reepithelialization, granulation tissue, and inflammatory response.

### Platelet endothelial cell adhesion molecule (PECAM-1), Vascular Endothelial Growth Factor (VEGF), and Proliferating Cell Nuclear Antigen (PCNA) by Immunohistochemistry

Some histological sections were used for immunohistochemistry of PECAM-1, VEGF, and PCNA^35, 45, 49^. For PECAM-1, sections were incubated with an anti-CD31 mouse monoclonal antibody (ab119339, abcam, Cambridge, UK) diluted at 1:100 in 3% BSA-PBS and incubated overnight at 4 °C. For VEGF, sections were incubated with an anti-VEGF rabbit polyclonal antibody (ab46154, abcam, Cambridge, UK) diluted at 1:100 in 1% BSA-PBS for 45 minutes. For PCNA, sections were incubated with an anti-PCNA rabbit polyclonal antibody (ab18197, abcam, Cambridge, UK) diluted at 1:4,000 in PBS for 2 hours.

All sections were incubated with a Mouse and Rabbit Specific HRP/DAB Detection IHC kit (abcam, Cambridge, UK) before counterstained with hematoxylin. Images were captured with a microscope imaging system. Images of the sections were quantified using ImageJ software (1.48 v, Wayne Rasband, National Institutes of Health, USA) with color threshold selection. The common values used for all samples in terms of Hue, Saturation, and Brightness are in the range between 50–235, 0–255, and 0–155, respectively. Once the area of the intensity with common values was selected, ImageJ calculated the area of intensity over background in percentage.

### C-reactive protein (CRP) by Enzyme-linked Immunosorbent Assay (ELISA)

Under general anesthesia, 1 ml of blood was collected from each rat’s heart through cardiac puncture before euthanasia. Each blood sample was then centrifuged at 9,500 rpm for 10 minutes. The serum was collected and diluted at 1:600,000. 50 ul of each sample was determined using a C-reactive protein (PTX1) rat ELISA kit (ab108827, abcam, Cambridge, UK).

### Statistical Analysis

All data were expressed as the mean ± standard deviation. Two-way analysis of variance (ANOVA) was used to analyze the main effects among the 4 groups and time points differences were analyzed with post-hoc Bonferroni tests. Student’s t-test for two independent samples was used for comparisons between groups of the same time point. Statistical analyses were performed using IBM SPSS 20.0 (IBM, Armonk, NY, USA), and statistical significance was considered at p < 0.05.

## Electronic supplementary material

Supplementary Information
