# Supplementary material for: Low-Magnitude High-Frequency Vibration Accelerated the Foot Wound Healing of n5-streptozotocin-induced Diabetic Rats by Enhancing Glucose Transporter 4 and Blood Microcirculation
Source: Sci Rep. 2017 Sep 14;7:11631. doi: 10.1038/s41598-017-11934-2 (PMC5599683; doi:10.1038/s41598-017-11934-2)
Supplement: Supplementary file 1 — Supplementary Information [file 41598_2017_11934_MOESM1_ESM.pdf]

Low-Magnitude High-Frequency Vibration Accelerated the Foot Wound Healing of n5-streptozotocin-induced Diabetic Rats by Enhancing Glucose Transporter 4 and Blood Microcirculation

1. Caroline Oi-Ling Yu, Msc.
   5/F Lui Che Woo Clinical Sciences Building, Department of Orthopaedics and Traumatology,

   Prince of Wales Hospital, The Chinese University of Hong Kong, Shatin, Hong Kong

   Email: Carolineyu@link.cuhk.edu.hk

2. Kwok-Sui Leung, MD.
   Orthopaedic Learning Centre, 1/F, Li Ka Shing Specialist Clinics, North Wing, Prince of Wales Hospital.

   Email: ksleung@cuhk.edu.hk

3. Jonney Lei Jiang, PhD.
   Rm509A, Lo Kwee-Seong Integrated Biomedical Sciences Building, School of Biomedical Sciences,The Chinese University of Hong Kong

   Email: heinzwg@163.com.

4. Tina Bai-Yan Wang, MMed.
   Rm509A, Lo Kwee-Seong Integrated Biomedical Sciences Building, School of Biomedical Sciences,The Chinese University of Hong Kong

   Email: b127748@cuhk.edu.hk

5. Simon Kwoon-Ho Chow, PhD.
   5/F Lui Che Woo Clinical Sciences Building, Department of Orthopaedics and Traumatology,

   Prince of Wales Hospital, The Chinese University of Hong Kong, Shatin, Hong Kong

   Email: skhchow@ort.cuhk.edu.hk

6. Wing-Hoi Cheung, PhD.*
   5/F Lui Che Woo Clinical Sciences Building, Department of Orthopaedics and Traumatology,

   Prince of Wales Hospital, The Chinese University of Hong Kong, Shatin, Hong Kong

   Tel: (852) 3505-1559; Fax: (852) 2632-4618;

   Email: louis@ort.cuhk.edu.hk

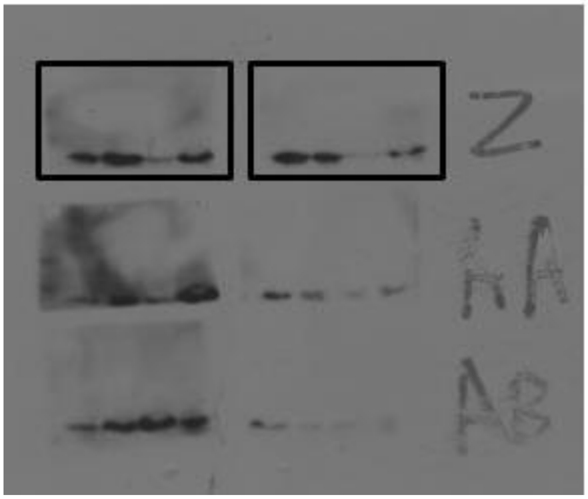

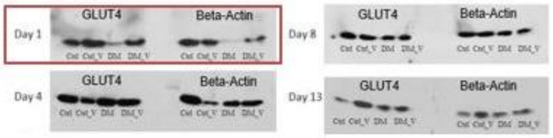

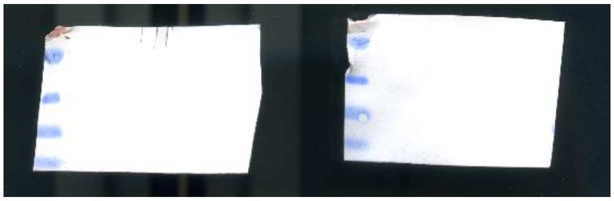

X-ray film of GLUT4 and beta-actin blots (aisle Z) at Day 1 post wounding. A scanned image of the molecular size markers on the PVDF membranes. Due to faint backgrounds on the developed x-ray films, the borders of each blot is approximated by its PVDF membrane.

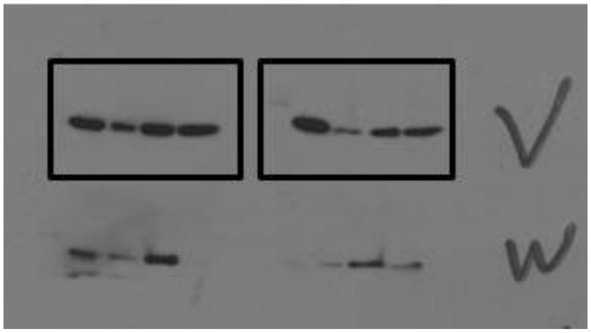

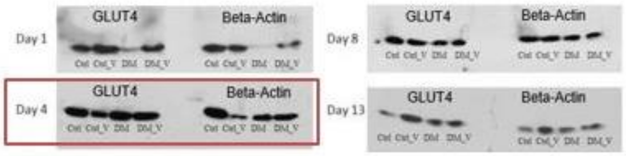

X-ray film of GLUT4 and beta-actin blots (aisle V) at Day 4 post wounding.

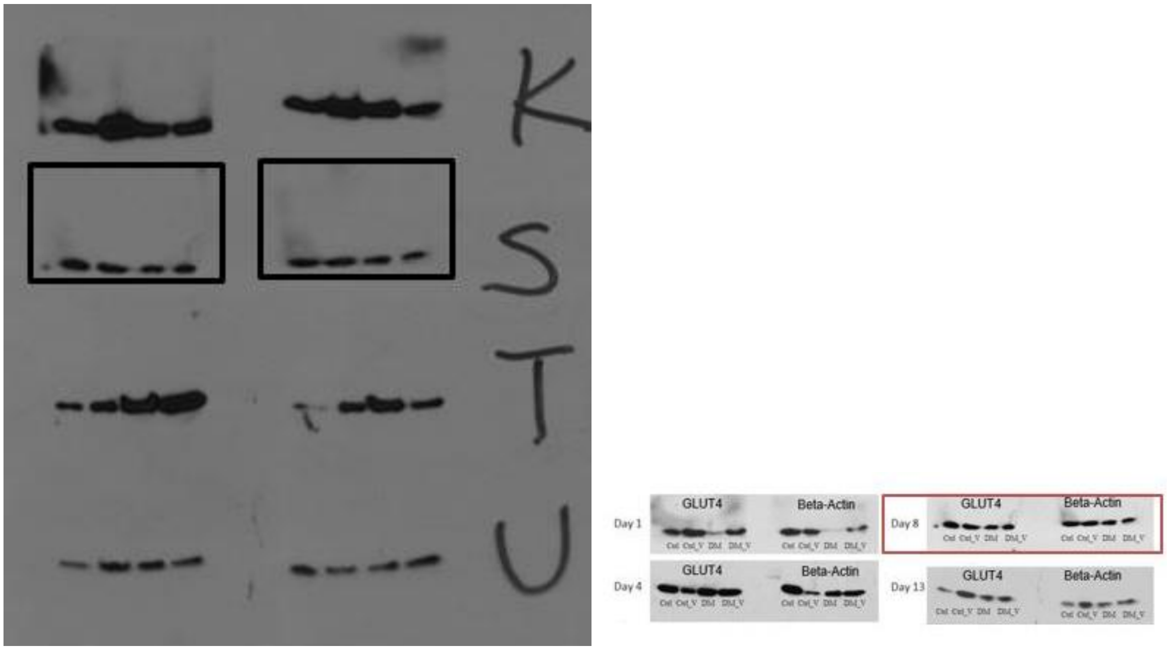

X-ray film of GLUT4 and beta-actin blots (aisle S) at Day 8 post wounding.

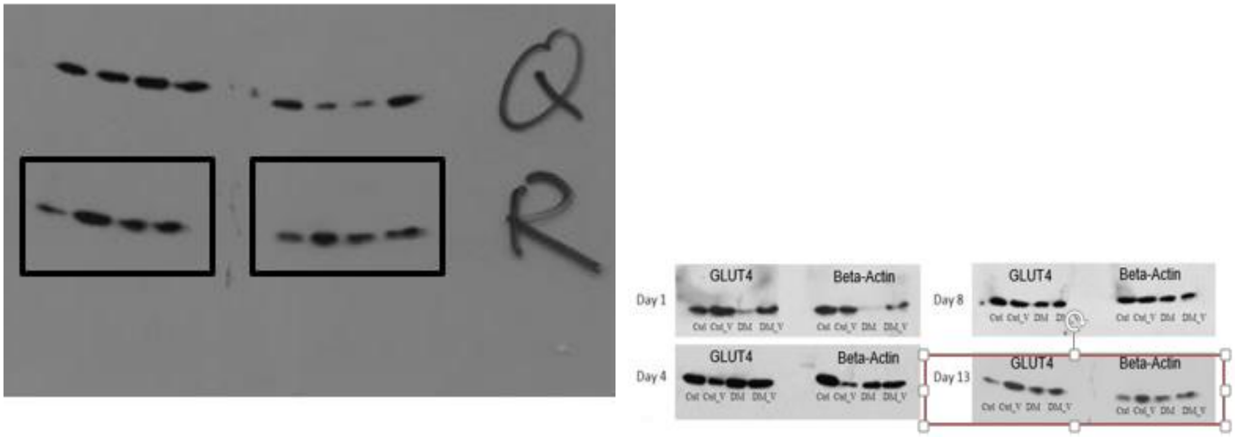

X-ray film of GLUT4 and beta-actin blots (aisle R) at Day 13 post wounding.
